# Multifaceted Roles of Asporin in Cancer: Current Understanding

**DOI:** 10.3389/fonc.2019.00948

**Published:** 2019-09-24

**Authors:** Shaohua Zhan, Jinming Li, Wei Ge

**Affiliations:** ^1^National Key Laboratory of Medical Molecular Biology, Department of Immunology, Institute of Basic Medical Sciences, Chinese Academy of Medical Sciences, Beijing, China; ^2^National Center for Clinical Laboratories, Beijing Hospital, National Center of Gerontology, Beijing, China; ^3^Affiliated Hospital of Hebei University, Baoding, China

**Keywords:** SLRP, aspirin, cell migration and invasion, metastasis, signaling pathways

## Abstract

The small leucine-rich proteoglycan (SLRP) family consists of 18 members categorized into five distinct classes, the traditional classes I–III, and the non-canonical classes IV–V. Unlike the other class I SLRPs (decorin and biglycan), asporin contains a unique and conserved stretch of aspartate (D) residues in its N terminus, and germline polymorphisms in the D-repeat-length are associated with osteoarthritis and prostate cancer progression. Since the first discovery of asporin in 2001, previous studies have focused mainly on its roles in bone and joint diseases, including osteoarthritis, intervertebral disc degeneration and periodontal ligament mineralization. Recently, *asporin* gene expression was also reported to be dysregulated in tumor tissues of different types of cancer, and to act as oncogene in pancreatic, colorectal, gastric, and prostate cancers, and some types of breast cancer, though it is also reported to function as a tumor suppressor gene in triple-negative breast cancer. Furthermore, asporin is also positively or negatively correlated with tumor proliferation, migration, invasion, and patient prognosis through its regulation of different signaling pathways, including the TGF-β, EGFR, and CD44 pathways. In this review, we seek to elucidate the signaling pathways and functions regulated by asporin in different types of cancer and to highlight some important issues that require investigation in future research.

## Introduction

**Figure 1:**
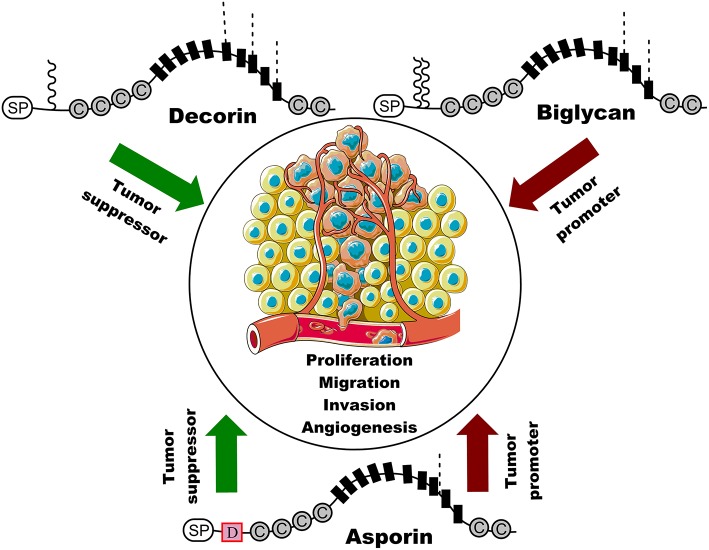
Schematic representation of the structure of three class I SLRP members and their roles in cancer. Decorin suppresses cancer cell proliferation, migration, invasion, and angiogenesis, whereas biglycan is positively associated with cancer cell proliferation, migration, invasion, and angiogenesis. Interestingly, asporin serves as tumor suppressor gene or oncogene in different types of cancer. SP indicates signal peptides and C represents cysteine region. Black boxes indicate a leucine rich repeat (LRR) motif and D represents the unique and conserved aspartic acid (D)-repeats in asporin. Waved line indicates O-linked glycosylation site and dashed line represents N-linked glycosylation. Original elements used in this diagram are from Servier Medical Art (http://smart.servier.com/).

Cancer is the leading cause of death from non-communicable disease, with an estimated 18.1 million new cases of cancer and 9.6 million cancer deaths, according to GLOBOCAN 2018 (1). Therefore, an in-depth understanding of tumor pathogenesis will be beneficial for the development of new pharmacological agents of therapeutic interventions, to decrease the global burden of cancer. The extracellular matrix (ECM) is a complex network of macromolecules with distinct physical and biochemical properties that participate in various cellular behaviors, including cell growth, survival, motility, and differentiation (2). Although tightly regulated in tissue development and homeostasis, the ECM influences the classical hallmarks of cancers, such as self-sufficient growth, insensitivity to growth inhibitors, evasion of apoptosis, limitless replicative potential, sustained angiogenesis, and tissue invasion and metastasis (3, 4). SLRP constitutes a major non-collagen component of the ECM and is ubiquitously distributed throughout the ECM in many tissues (5). Similarly, SLRP is also involved in various pathological processes resulting in skin fragility, cardiovascular disease, osteoporosis, osteoarthritis, and cancer (6–9). The SLRP family consists of 18 members categorized into five distinct classes: the traditional classes I-III and the non-canonical classes IV-V. This categorization is based on N-terminal cysteine-rich clusters, core leucine-rich repeats (LRR), C-terminal ear repeat motifs, and genomic organization (10, 11). Most SLRP proteins are proteoglycans containing chondroitin/dermatan sulfate or keratan sulfate chains, while others are glycoproteins containing N-linked oligosaccharides (9). SLRPs have been shown to interact with various extracellular receptors or ligands through their bare β-sheets present on the concave surface of LRR, such as collagens, fibronectin, bone morphogenic protein-4 (BMP-4), and transforming growth factor-β1 (TGF-β1) (12–14). This interaction then involves several signaling pathways that regulate the cell-matrix function. Class I SLRPs, which have the highest homology (~50% identity) based on the amino acid sequence, contains three classical members: decorin, biglycan, and asporin (9, 15). These three class I members contain 10 LRRs and are distinguished by a unique cysteine-rich cluster in the N terminus consensus (CX_3_CXCX_6_C). The N-terminal regions of decorin and biglycan carry one and two chondroitin/dermatan sulfate chains, respectively (9, 15, 16). Decorin is a natural receptor tyrosine kinase (RTK) inhibitor that functions through binding the epidermal growth factor receptor (EGFR), insulin like growth factor-1R (IGF-1R), fibroblast growth factor receptor (FGFR), and c-met. Thus, decorin blocks several biological processes, such as cell growth, cell evasion, and migration, through the induction of p21 via EGFR and downregulation of the c-met/β-catenin/myc pathway (17). Furthermore, decorin also modulates cancer through its interaction with TGF-β (18). Therefore, decorin is regarded as the “endogenous guardian” of the matrix, due to its anti-proliferative, anti-metastatic, and angio-suppressive effects. In contrast, biglycan acts as a danger signal by affecting both immune responses and tumor characteristics (17, 18). Various studies have shown that the upregulation of biglycan in cancer stroma is positively correlated with cell proliferation, migration, metastasis, and angiogenesis through the regulation of the TLR/NF-κB, MAPK, and the FAK signaling pathway (18). It has also been demonstrated that high biglycan expression is positively associated with pro-malignant potential and poor patient prognosis (19, 20). However, asporin exerts negative and positive roles in the pathogenesis and prognosis of different cancers (Figure 1). Therefore, we will narrow the focus of this review to asporin, simply describing its sequence, structure, and functions, and primarily highlighting its multifaceted roles in cancers. Although asporin has been reported using another name PLAP-1 (21), the term asporin is used exclusively in this review.

## Sequence, Structure, and Functions of Asporin

Asporin was initially identified as an extracellular secreted protein in human articular cartilage or periodontal tissue by three independent groups in 2001 (15, 16, 21). The name “asporin” refers to the unique aspartic acid residues in its N terminus and its similarity to decorin. The human *asporin* gene has eight exons and spans 26 kilobases on chromosome region 9q22.31 (16). Asporin protein consists of 380 amino acids and its amino acid sequence is 54%/60% identical to decorin and biglycan, respectively. However, compared to decorin and biglycan, asporin cannot be considered as a proteoglycan in the strictest sense because it lacks the serine/glycine dipeptide sequence for O-linked glycosaminoglycan binding. Furthermore, unlike other proteoglycans, asporin contains a unique and conserved stretch (8–19) of aspartate residues (D-repeat) in its N terminus (12, 22). Two studies have demonstrated that asporin D14 variants increase susceptibility to and severity of knee osteoarthritis in Japanese and Chinese Han populations, whereas D13 was found to be significantly protective against osteoarthritis in some Japanese populations (12, 23). However, these findings were not confirmed in other populations in the United States (24), Spain (25), and Iran (26). Therefore, the relationship between asporin polymorphisms and osteoarthritis still needs to be investigated in large-scale studies of different ethnic populations.

The pathogenesis of osteoarthritis is characterized by an imbalance between the degradation and synthesis of the cartilage ECM, with type II collagen and aggrecan being the primary components that play critical roles in the viscoelasticity and tensile strength of cartilage (27). The same research group indicated that amino acids 159–205 of asporin interact directly with TGF-β1 and, compared with other alleles, its D14 allele significantly inhibits TGF-β1-induced expression of genes including type II collagen and aggrecan (12, 28). Furthermore, this inhibition is due to asporin blockade of TGF-β1 binding to its receptor TβRII (29). Interestingly, TGF-β1 indirectly induces an asporin expression at both the mRNA and protein levels through its downstream Smad pathway, particularly involving Smad3 (29, 30). Therefore, asporin and TGF-β1 form a functional feedback loop in cartilage and play vital roles in homeostasis and the pathogenesis of osteoarthritis. There is an additional regulatory feedback loop between asporin and BMP-2, which is also correlated with the severity of osteoarthritis (31, 32). In 2007, Yamada et al. demonstrated that asporin co-localize with BMP-2 *in vitro* (33). Previous studies also indicate that asporin acts as a negative regulator of cytodifferentiation and mineralization by regulating BMP-2 activity (33), and that asporin D14 inhibits BMP-2 signal transduction more efficiently than D13 (34). Conversely, BMP-2 also upregulates asporin mRNA and protein expression (35). As for the TGF-β1–mediated cartilage matrix gene, type I and type II collagen also bind to asporin (28, 36). Furthermore, the D-repeat domain of asporin interacts with calcium to stimulate the biomineralization of collagen. It may appear intriguing that, unlike biglycan, another class I SLRP member, decorin, inhibits asporin-induced collagen mineralization (36). Thus, although asporin shows significant associations with osteoarthritis, functional differences among D-repeat polymorphisms in asporin are still unclear. It is speculated that asporin D-repeat-length may influence conformational changes that consequently alter processes such as BMP-2 signaling, TGF-β1 signaling, and collagen mineralization, although further studies are required to clarify the underlying molecular mechanisms. Nevertheless, all these previous studies have a good implication for the following cancer research, which will be discussed in detail.

## Emerging Roles of Asporin in Cancer

Bioinformatics analysis of microarray data indicate the potential of asporin as a biomarker for colorectal cancer detection and prevention (37). Recently, two studies also demonstrated that asporin is a potential biomarker in gastric cancer based on the integrated analysis of gene expression profiles (38, 39). Turtoi et al. found that asporin was upregulated in pancreatic ductal adenocarcinoma (PDAC) tissues compared to the corresponding normal tissues based on proteomics analysis and confirmed by immunohistochemistry (40). A recent report by Klee and colleagues indicated that serum asporin was upregulated in men with advanced prostate cancer (41). Furthermore, two studies demonstrated that asporin was not only elevated in invasive ducal breast carcinoma compared to ductal carcinoma *in situ* but also responded to aromatase inhibitor treatment (42, 43). All these studies suggest that asporin plays vital roles in the pathogenesis of different types of cancer, and a considerable amount of research has indicated that asporin acts as an oncogene in pancreatic (44), colorectal (45, 46), gastric (47, 48), and prostate cancer (49), as well as some types of breast cancer (50–52), but as a tumor suppressor gene in triple-negative breast cancer (52) via different signaling pathways. Therefore, here, we seek to elucidate the signaling pathways and different functions regulated by asporin in different types of cancer and to highlight some important issues that still need to be investigated.

## Cancer-Related Pathways Regulated by Asporin

**Figure 2:**
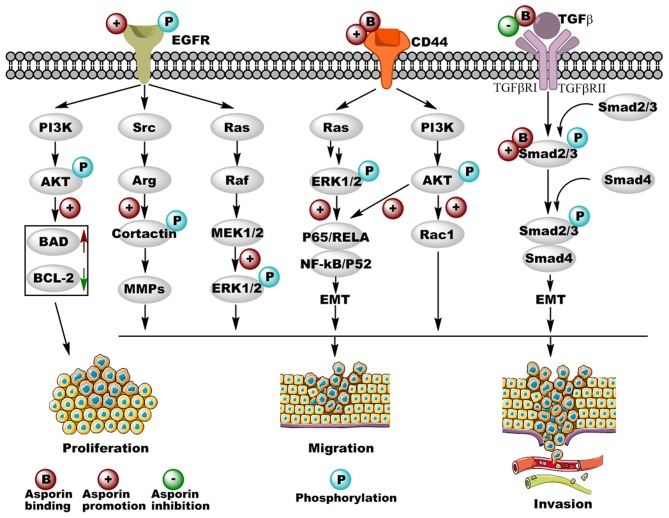
Cancer-related pathways regulated by asporin in different types of cancer. Asporin not only promotes cancer cell proliferation, migration, and invasion by binding with CD44 and Smad 2/3 as well as promoting the phosphorylation of EGFR, but also inhibits cancer cell migration and invasion by binding with TGF-β in the extracellular matrix. Upregulated and downregulated proteins are shown in solid red and green arrows, respectively. Original elements used in this diagram are from Servier Medical Art (http://smart.servier.com/).

Numerous studies have indicated that deregulated signaling pathways result in proliferation, invasion, and metastasis of cancer cells. The most significant cancer-related pathways regulated by asporin are TGF-β, EGFR, and CD44 signaling pathways; altered expression of components of these signaling pathways and the regulatory roles of asporin are illustrated in Figure 2.

## TGF-β Signaling

The TGF-β/Smad2/3 signaling pathway plays critical roles in cancer cell behavior through the unique TGF-β serine-threonine kinases and exerts both tumor suppressor and promoter activity in tumor progression and invasion. Furthermore, TGF-β secreted by tumor cells acts not only on elements of the tumor microenvironment in a paracrine fashion, but also on the tumor cell itself via autocrine effects (53, 54). In the early stages of breast cancer, TGF-β1 shows anti-tumor activity by mediating growth arrest and cancer cell apoptosis; however, in the late stages, TGF-β1 enhances the malignancy of breast cancer cells (55). Previous studies have demonstrated that asporin interacts directly with TGF-β1 and inhibits downstream gene expression of aggrecan and collagen in osteoarthritis (12). In breast cancer, Maris et al. reported that asporin was upregulated in the stroma of breast cancer lesions but not in normal tissues, indicating that asporin influences the tumor microenvironment. This group also found that asporin expression was promoted by TGF-β1 and inhibited by IL-1β in normal breast fibroblasts, as well as cancer-associated fibroblasts (CAFs) (52). Furthermore, asporin inhibited triple-negative breast tumor growth and metastasis *in vivo*, via a molecular mechanism in which asporin may interact with TGF-β1 to inhibit its downstream Smad2 activation, resulting in the suppression of epithelial-to-mesenchymal transition (EMT) and stemness in triple-negative breast cancer cells (52). Similarly, dysregulated TGF-β signaling pathway plays pivotal roles in the development of colorectal cancer (56). Li et al. indicated that asporin enhances cell growth, migration, and invasion via activation of the TGF-β/Smad2/3 signaling pathway in colorectal cancer (45). Experimental evidence revealed that asporin interacts directly with Smad2/3 and facilitates the entry of p-Smad2/3 into the nucleus, which induces EMT and colorectal cancer progression (45). This was the first study to show the function of asporin as an intracellular molecule and not as an extracellular matrix component in cancer. Altogether, these results indicate that asporin binds directly to extracellular TGF-β1 or cytoplasmic Smad2/3, resulting in the inhibition or activation of the TGF-β signaling pathway, respectively. It is therefore not surprising that under different conditions, asporin acts as a tumor suppressor gene in triple-negative breast cancer and as an oncogene in colorectal cancer.

## EGFR Signaling

EGFR is a tyrosine kinase receptor that drives many types of epithelial tumors, including metastatic colorectal cancer, non-small-cell lung cancer, pancreatic cancer, and breast cancer (57). Aberrant activation of the EGFR signaling pathway is critical for cancer cell apoptosis, proliferation, differentiation, and motility via the downstream RAS/RAF/MEK/ERK and AKT/PI3K/mTOR pathways (57, 58), and tremendous amounts of research have implicated EGFR as a potential target for cancer therapy (59). Upregulated expression and activation of EGFR are correlated with tumor invasion and poor prognosis, indicating that the EGFR signaling pathway is also critical in gastric cancer (60). In 2015, Ding et al. found that asporin promotes the activation of p-EGFR and its downstream p-ERK1/2 but not their corresponding total proteins (48). Small inhibitory RNA-mediated silencing of asporin in gastric cell lines not only inhibits cell proliferation and survival through the downregulation of anti-apoptotic Bcl-2 and the upregulation of pro-apoptotic Bad, but also blocks cell migration by the downregulation of the EGFR/ERK/MMP2-mediated signaling axis (48). As recently shown, Zhang et al. found that asporin is also located in the cytoplasm and nuclei of gastric cancer cell lines and could promote their proliferation. The underlying mechanism is that asporin interacts with PSMD2 and enhances PSMD2 mediated degradation of tumor suppressor factors (DUSP7, WIP1, and PTEN), resulting in the activation of MAPK/ERK, P38/MAPK, and PI3K/AKT signaling pathways (61). In colorectal cancer, Wu et al. suggested that asporin promotes cancer cell endothelial tube formation by upregulating VEGF expression (46). Furthermore, asporin has been shown to facilitate colorectal cancer cell migration and invasion by successively activating p-EGFR^Tyr1173^, p-Src^Tyr416^, and p-Cortactin^Tyr421^ (46), which is important for the formation of invadopodia and secretion of matrix metalloproteinases (MMPs) (62, 63). These results indicate that asporin-mediated EGFR/Src/Cortactin signaling is critical for colorectal cancer metastasis. Therefore, EGF and PP2 (Src inhibitor) inhibit the activation of the EGFR/Src/Cortactin pathway mediated by asporin (46). However, the molecular mechanism by which asporin activates the EGFR signaling pathway in gastric and colorectal cancer remains to be investigated both *in vitro* and *in vivo*.

## CD44 Signaling

CD44 is a non-kinase transmembrane glycoprotein that exerts its cellular functions via interactions with several ligands, including hyaluronic acid (HA), osteopontin (OPN), collagen, and MMPs (64). By binding with CD44, HA induces conformational changes leading to adaptor protein recruitment to the intracellular cytoplasmic tail of CD44 and the subsequent activation of various signaling pathways involved in tumor progression (65). CD44 is involved in several types of cancers, including pancreatic, colorectal, breast, and prostate cancer, as well as head and neck squamous cell carcinoma, and gastrointestinal cancer (66). Furthermore, CD44 regulates tumor progression, metastasis, angiogenesis, and chemoresistance by activation of different cytoskeletal changes and signaling pathways, including MAPK, Hippo, β-catenin, AKT, TGF-β, MMPs, and STAT3 (66). In scirrhous gastric cancer, asporin is also an important ligand of CD44 (47). Satoyoshi et al. indicated that asporin was primarily expressed in cancer stroma but was not observed in normal tissues (47), which is consistent with the patterns of expression in pancreatic and breast cancer (44, 52). Experimental evidence shows that the gastric cancer cell line 44As3 promotes asporin expression in CAFs via a mechanism in which, asporin as a unique class I SLRP, enhances the co-invasion of CAFs and cancer cells both *in vitro* and *in vivo* through the CD44/Rac1 mediated axis (47). In pancreatic cancer, asporin was also shown to interact directly with CD44 in co-precipitation assays (44), and asporin not only facilitated cancer cell migration and invasion *in vitro* but also enhanced tumor metastasis *in vivo*. Although the binding motifs are unclear, asporin-CD44 binding is known to activate the CD44/AKT/NF-κB/p65 and CD44/ERK/NF-κB/p65 axes to promote EMT in a paracrine/autocrine pattern, resulting in the upregulated expression of ZEB1, N-catenin, vimentin, slug, and snail as well as the downregulated expression of ZO-1 and E-cadherin. Therefore, from a molecular perspective, the asporin/CD44/EMT signaling pathway could be considered as a potential therapeutic target axis to decrease tumor migration and invasion in pancreatic and gastric cancer.

## Future Potential Directions of Asporin Mediated Signaling Pathways in Cancer

Recently, Hughes and co-workers found that asporin could not only sustain the self-renewal capacity of the mesenchymal stromal cell but also restrict early mesenchymal stromal cell differentiation via inhibiting the BMP-4-induced signaling pathway (67). Furthermore, in asporin null mice, they also found that there are decreased tumor-associated mesenchymal stromal cells, fewer cancer stem cells, reduced tumor vasculature, and increased infiltrating CD8^+^T cells in the prostate tumor allografts (67). All these results indicate that asporin is a critical regulator in the tumor microenvironment possibly by regulating different signaling pathways except for TGF-β, EGFR, and CD44 pathways, and we can get some clues from previous studies not associated with cancer. As a secreted extracellular protein, it has been demonstrated that asporin could interact with several ligands as well as with surface receptors, including BMP-2, BMP-4, FGF-2, WNT8, Nodal, IGF, and IGF1R (33, 67–69). Whether asporin could interact with these proteins and regulate the corresponding signaling pathways in cancer needs to be investigated in the future. Furthermore, the previous review indicated that SLRP could affect several RTKs, including the ErbB family, the hepatocyte growth factor receptor (Met) and IGF1R (70). Thus, we wonder whether asporin could regulate Met and ErbB2 signaling pathways in cancer, especially in breast cancer. As an intracellular protein, asporin could interact with PSMD2, which is responsible for substrate recognition and binding (61). Therefore, whether asporin could mediate another intracellular substrate proteasomal degradation via binding with PSMD2 in different types of cancer, still needs to be uncovered. Collectively, because asporin could bind additional and currently unidentified proteins to regulate different signaling pathways in cancer, it is an interesting direction to globally screen additional asporin-interactive partners through quantitative (measuring dissociation constants) and proteomics (identifying interacting proteins) analyses.

## Perspectives on the Potential Role of Asporin in the Diagnosis and Prognosis of Cancer

From a clinical perspective, Maris et al. demonstrated that the areas under the curves (AUC) of asporin, to discriminate breast cancer patients with different outcomes was 0.87, and that low asporin expression is significantly correlated with reduced overall survival (52). However, another study showed that asporin has a dual role in the progression of breast cancer. In 2016, Simkova et al. demonstrated that a high expression of asporin correlated with good relapse-free survival (RFS) in grades I/II in breast cancer patients, but was associated with worse RFS in grade III patients regardless of tumor ER status (51). The dual role of asporin in breast cancer progression may be due to its D-repeat polymorphism, which has been described in prostate cancer progression (22); however, this hypothesis needs to be further investigated in large-scale population studies. Recently, elevated asporin gene expression was shown to be significantly correlated with worse overall survival and disease-free survival in gastric cancer (39). In colorectal cancer, high asporin expression showed a positive relationship with lymph node metastasis and high TNM stage, but not with sex, age and tumor size (46). Furthermore, upregulated asporin expression was correlated with worse overall and disease-free survival, and was implicated as an independent indicator of a worse prognosis through a multivariate analysis (45). In pancreatic cancer, asporin is mainly expressed in the cancer stroma, but only in cancer cells in a small proportion of patients (44). Furthermore, high asporin expression in the cancer stroma is positively correlated with poor overall survival, while there is no relationship between asporin expression in cancer cells and clinical outcome (44). Similarly, asporin is primarily expressed in the tumor stroma in prostate cancer, but not in benign tissue (22, 49). Interestingly, asporin is also positively associated with the presence of a reactive stroma (49), which is associated with disease progression and mortality in prostate cancer (71). Furthermore, two studies also demonstrated that elevated expression of asporin mRNA or protein was correlated with biochemical recurrence and higher Gleason score in independent prostate cancer cohorts (22, 49). A multivariable Cox proportional hazard analysis indicated that asporin expression in the stroma was an independent prognostic factor for biochemical recurrence (49). Moreover, Hurley et al. suggested that homozygous germline asporin D14 and heterozygous D13/14 were significantly associated with lymph node involvement and metastatic recurrence in prostate cancer, whereas homozygous D13 was significantly protective against metastatic recurrence in a multivariable analysis (22). Additionally, in an orthotopic xenograft model, co-injection of overexpressed asporin D14 fibroblast and PC-3 cancer cells increased the number of metastases to lymph nodes and other organs, including lung, liver, and pancreas compared to asporin D13, although the underlying molecular mechanisms are still unclear (22). Our current understanding of the dual role of asporin in cancer diagnosis and prognosis is summarized in Table 1.

**Table 1:** Experimental evidence of asporin expression in human malignancies.

**Cancer types**	**Asporin expression**	**Cell lines/models**	**Outcome**	**Clinical functions**	**References**
Breast cancer	Up-regulation in tumor stroma	NBF/CAF cells MCF-7/T47D/ZR751/SKBR3/BT-474/MB-231/MB-468/BT-549/MCF-10A cell lines NOD-SCID mouse model	Inhibiting EMT transition, and stemness *in vitro* Reducing growth and metastasis *in vivo*	(+) good outcome (+) increased overall survival	(52)
Breast cancer	Up-regulation in tumor stroma and tumor cells	CAF cells Hs578T/MDA-MA-231/BT549/T47D cell lines PyMT mouse model	Promoting invasion *in vitro*	(+) better relapse free survival in low-grade patients (+) worse relapse free survival in high-grade patients	(50, 51)
Prostate cancer	Up-regulation in tumor stroma and blood	CAF/EPF/PrSC cells PC-3/LNCaP cell lines p53 null mouse model	(+) aggressive prostate cancer (+) neuroendocrine marker expression	(+) higher biochemical recurrence (+) higher Gleason score (+) reactive stroma	(41, 49)
Prostate cancer (asporin D13/14 or D14)	Up-regulation in tumor stroma	CAF cells WPMY1/PC3 cell lines NSG mouse model	(+) distant lymph nodes (+) distant organ metastasis	(+) worse metastasis free survival (+) lymph node involvement (+) higher Gleason score	(22)
Gastric cancer	Up-regulation in tumor stroma	NF/CAF cells 44As3/GES/SGC-7901/BGC-823/AGS/MKN45/N87 cell lines BALB/c nude mouse model	Promoting survival, proliferation, migration, and invasion *in vitro* Promoting invasion *in vivo*	(+) worse overall survival (+)worse disease-free survival	(39, 47, 48, 61)
Colorectal cancer	Up-regulation in tumor stroma and tumor cells	CAF cells HCT-8/RKO/HT-29/LoVo /Caco2 /HCT116/SW1116/ SW480/RKO/SW620 cell lines BALB/c nude mouse model	Promoting EMT transition, cell viability, migration, invasion, and endothelial tube formation *in vitro* Promoting liver metastasis *in vivo*	(+) higher TNM stage (+) lymph node involvement (+) worse overall survival (+) worse disease-free survival	(45, 46)
Pancreas cancer	Up-regulation in tumor stroma	PSC/CAF cells AsPC-1/BxPC-3/MIA PaCa-2/PANC-1 cell lines Nude mouse model	Promoting EMT transition, migration, and invasion *in vitro* Promoting tumor invasion *in vivo*	(+) worse overall survival	(44)

*EMT, epithelial-to-mesenchymal transition; TNM, TNM classification of malignant tumors; EPF, fetal prostate fibroblasts; PrSC, primary prostate stromal cells; CAF, cancer associated fibroblast; PSC, pancreatic stellate cells*.

## The Therapeutic Potential of Asporin Modulation in Cancer

Asporin could enhance the proliferation, migration, and invasion capacity of pancreatic, colorectal, gastric, prostate, and breast cancer cells (44–50, 61, 67), indicating it could be regarded as a valuable therapeutic target. Although non-drugs are currently in clinical trials for the treatment of patients with asporin dysregulation cancers, there are several potential strategies to reduce asporin functional dose in future cancer studies: (1) inhibition of asporin protein-protein interactions; (2) targeting *asporin* mRNA. Previous studies indicate that asporin could interact with CD44, TGF-β, BMP-2, BMP-4, FGF-2, WNT8, Nodal, IGF, and IGF1R in different microenvironments to regulate different signaling pathways. Therefore, peptide antagonists derived from asporin or its interaction partners may block asporin protein-protein interactions to inhibit corresponding signaling pathways. Additionally, the application of RNAi strategies are potential approaches to decrease asporin translational level, including antisense oligonucleotides, short interfering RNA, and short hairpin RNA. Furthermore, a revolutionary gene-editing tool CRISPR/Cas9 could be explored to deplete asporin expression in a tissue-specific manner (72). As asporin exerts tumor suppression in triple-negative breast cancer (52), increasing the asporin function dose may also be an anti-cancer strategy. Previous studies demonstrated that IL-1β, miR-21, and miR-101 could downregulate the asporin protein and mRNA level, respectively (52, 73). Antisense oligonucleotides toward IL-1β, miR-21, and miR-101 may be useful therapeutic applications when asporin acts as a tumor suppressor. In pre-clinical studies, different types of models could be valuable in order to test these potential therapeutic strategies in regulating asporin expression, including xenograft mouse models, allograft mouse models, genetically engineered mouse models, patient-derived models (PDX), and PDX 3D spheroids (67, 74). Altogether, further studies, especially mouse models and clinical trials, are needed to investigate the therapeutic potential of asporin modulation in cancer.

## Conclusions

Class I SLRP members are ubiquitously distributed in the ECM of many tissues and play critical roles in tumor proliferation, migration, invasion, and angiogenesis. Although decorin is regarded as the “endogenous guardian” and biglycan acts as a danger signal in cancer, asporin acts as an oncogene in some types of cancer (breast, pancreatic, colorectal, gastric, and prostate), but as a tumor suppressor gene in triple-negative breast cancer (75). Since the first discovery of asporin in 2001, studies have focused mainly on its role in bone and joint diseases, including osteoarthritis, intervertebral disc degeneration, and periodontal ligament mineralization (76, 77). Recently, asporin expression was also shown to be dysregulated in tumor tissues and positively or negatively correlated with tumor proliferation, migration, invasion, and patient prognosis by regulating different signaling pathways, including the TGF-β, EGFR, and CD44 pathway. However, various important issues associated with asporin in cancer remain to be investigated in future studies. First, asporin contains a unique and conserved stretch of aspartate residues in its N terminus, and germline polymorphisms in D-repeat-length are associated with osteoarthritis and prostate cancer progression; however, functional differences and the molecular mechanisms underlying the influence of different D-repeat polymorphisms remain to be clarified. Second, although asporin is primarily expressed in the ECM, asporin expression is also observed in the cytoplasm and nucleus (45, 46, 50, 61). The biological function of asporin inside cancer cells was largely neglected until it was found that asporin interacts with intracellular Smad2/3 and PSMD2 to facilitate gastric and colorectal cancer progression. Thus, the intracellular function of asporin in cancer and whether asporin subcellular localization is controlled by its post-translational modifications, still needs to be investigated. Third, as TGF-β, EGFR, and CD44 pathways play vital roles in other types of cancer, including head and neck cancer, gall bladder cancer, glioblastoma, lung cancer, gastrointestinal cancer, and so on, whether asporin is an oncogenic driver or tumor suppressor in these cancers still need to be investigated in the future. Finally, due to the function of asporin as a tumor suppressor gene and oncogene in different types of cancer, the exact molecular mechanisms of its dual role in different tumor microenvironments remain to be elucidated. Overall, only dedicated studies that investigate the molecular mechanisms underlying the roles of asporin in cancer will pave the way for the development of new pharmacological agents for therapeutic interventions.

## Author Contributions

SZ prepared the table and figures. SZ, JL, and WG wrote, read, and approved the final manuscript.

### Conflict of Interest

The authors declare that the research was conducted in the absence of any commercial or financial relationships that could be construed as a potential conflict of interest.
